# Bacterial Community of Grana Padano PDO Cheese and Generical Hard Cheeses: DNA Metabarcoding and DNA Metafingerprinting Analysis to Assess Similarities and Differences

**DOI:** 10.3390/foods10081826

**Published:** 2021-08-07

**Authors:** Miriam Zago, Lia Rossetti, Tommaso Bardelli, Domenico Carminati, Nelson Nazzicari, Giorgio Giraffa

**Affiliations:** 1 Research Centre for Animal Production and Aquaculture (CREA-ZA), Council for Agricultural Research and Economics, 26900 Lodi, Italy; miriam.zago@crea.gov.it (M.Z.); lia.rossetti@crea.gov.it (L.R.); domenico.carminati@crea.gov.it (D.C.); nelson.nazzicari@crea.gov.it (N.N.); 2 Research Centre for Plant Protection and Certification (CREA-DC), Council for Agricultural Research and Economics, 26900 Lodi, Italy; tommaso.bardelli@crea.gov.it

**Keywords:** Grana Padano cheese, generical hard cheeses, bacterial diversity, DNA metabarcoding, DNA (meta)fingerprinting, predictive models, neural network

## Abstract

The microbiota of Protected Designation of Origin (PDO) cheeses plays an essential role in defining their quality and typicity and could be applied to protect these products from counterfeiting. To study the possible role of cheese microbiota in distinguishing Grana Padano (GP) cheese from generical hard cheeses (HC), the microbial structure of 119 GP cheese samples was studied by DNA metabarcoding and DNA metafingerprinting and compared with 49 samples of generical hard cheeses taken from retail. DNA metabarcoding highlighted the presence, as dominant taxa, of *Lacticaseibacillus rhamnosus*, *Lactobacillus helveticus*, *Streptococcus thermophilus*, *Limosilactobacillus fermentum*, *Lactobacillus delbrueckii, Lactobacillus* spp., and *Lactococcus* spp. in both GP cheese and HC. Differential multivariate statistical analysis of metataxonomic and metafingerprinting data highlighted significant differences in the Shannon index, bacterial composition, and species abundance within both dominant and subdominant taxa between the two cheese groups. A supervised Neural Network (NN) classification tool, trained by metagenotypic data, was implemented, allowing to correctly classify GP cheese and HC samples. Further implementation and validation to increase the robustness and improve the predictive capacity of the NN classifier will be needed. Nonetheless, the proposed tool opens interesting perspectives in helping protection and valorization of GP and other PDO cheeses.

## 1. Introduction

Protected Designation of Origin (PDO) products represent the excellence of European agricultural food production and are the result of the interplay between environmental (e.g., climate) and human factors (e.g., production techniques handed down over time), which are typical of a given territory. To this regard, many cheeses benefit from the PDO quality label. PDO cheeses are subject to specific production conditions, which producers spontaneously adhere to by joining the Consortia, which establish the transformation criteria through specific shared rules and guidelines. In this way, the consumer is guaranteed in terms of transparency and traceability, while benefiting from high-quality products [1]. The overall quality of cheeses, including PDO cheeses, is the result of many concomitant factors, such as the quality of the raw material, the farming methods, and the processing technology, which, in some cheeses (such as Grana Padano, Parmigiano Reggiano, Silter, Pélardon, Poro), involves the use of undefined microbial cultures. The interaction between these elements contributes to shape the qualitative and quantitative microbiological content of the ripened product, giving it a specific composition [2,3,4,5,6,7,8]. On the other hand, the increasing frequency of imitations and frauds involving PDO foods, including cheeses [9], addressed the search for finer and more sensitive methods of discrimination.

In 2020, 210,000 tons (or approximately 5,200,000 cheeses) of Grana Padano (GP) were produced. With a growing export trend (40% of total production, +3.3% compared to 2019), GP is increasingly consumed both in EU and non-EU countries (https://www.granapadano.it, accessed on 5 July 2021) [10]. Grana Padano is a cooked, long ripened PDO cheese made from raw and partially skimmed milk, which is fermented by lactic bacteria present in natural starters (‘sieroinnesto’). The sieroinnesto, which is one of the typical elements of Grana Padano, is prepared by backslopping, i.e., using part of the drained whey from the previous day’s cheese making, which is left for 18–24 h at 42–45 °C until a final acidity of approx. 60 °SH/100 (pH 3.3–3.6) is reached [2,11,12]. On the other hand, GP is also one of the most imitated and counterfeited cheeses. This has stimulated the search for increasingly sensitive analytical approaches to GP mapping and characterization. A recent investigation carried out on a limited number of cheese samples using untargeted metabolomics revealed differences in chemical fingerprints between PDO and non-PDO grana-like cheeses [13]. In other studies, geographic or technological differences were observed in the microbiota of cheese, suggesting it as a possible tool to establish cheese authenticity and diversity [14,15,16,17]. In a previous study, the bacterial taxa present in GP were highlighted by 16S rRNA gene sequencing (DNA metabarcoding) [18]. In the present study, the usefulness of the cheese microbiota to distinguish GP cheese from generical hard cheeses, i.e., cheeses whose appearance could be confused with Grana Padano PDO cheese, was investigated. The structure and the genotypic fingerprinting of the bacterial taxa of 119 GP samples were evaluated by 16S rRNA gene sequencing (DNA metabarcoding) and RAPD-PCR from total cheese DNA (RAPD-PCR metafingerprinting), respectively, and compared with 49 samples of generical hard cheeses retrieved from retail.

## 2. Materials and Methods

### 2.1. Sampling of Cheeses

One hundred nineteen samples (2-kg slice each), representative of all producers of GP and the entire geographical area of production, were collected. Cheeses had been produced in August 2018 and had a ripening time of 6–7 months. Forty-nine samples of generical hard cheeses (from now on ‘HC’), labelled as EU and extra-EU products, were retrieved from retail. All cheese samples were stored at −20 °C. After thawing at 4 °C for 18 h, slices (10 g each) including three different sections of the cheese (i.e., outer, central, and inner) were sampled and put into sterile containers.

### 2.2. Total DNA Extraction

The 10-g cheese slices were treated as described by Zago et al. [18]. Briefly, the samples were homogenized twice in a Stomacher 400 Circulator (Seward Laboratory, London, UK) with sterile sodium citrate (NaCt 2% *w*/*v*, pH 7.5). The cheese homogenate was centrifuged (14,400× *g* for 7 min at 4 °C) for fat removing. The pellet was then resuspended in Triton X-100 (2.5% *v*/*v*), washed twice with phosphate-buffered saline (pH 7.5), and centrifuged (8700× *g*, 7 min, 4 °C). Finally, after the pellet was resuspended in Tris-EDTA (0.1 M, pH 8), total dsDNA was extracted using a QIAcube HT automated station (Qiagen, Milan, Italy) using QIAamp 96 QIAcube HT kit (Qiagen, Milano, Italy). Total dsDNA was quantified fluorometrically (Qubit^TM^, Life Technologies, Monza, Italy).

### 2.3. DNA Metabarcoding

Total DNA extracted from the 119 GP and the 49 HC samples was subjected to DNA metabarcoding analysis (IGATech, Udine, Italy) by sequencing of the variable V3–V4 regions of the 16S rRNA gene using an Illumina MiSeq platform, as described previously [18].

### 2.4. RAPD-PCR Metafingerprinting

RAPD-PCR from total DNA of each sample was carried out according to Zago et al. [18]. PCR products were separated by QIAxcel electrophoresis using dedicated DNA Screening Gel Cartridges (Qiagen, Milan, Italy). Two QX Alignment Markers (15 bp–5 kb and 15 bp–600 bp, in a 1:1 ratio) and the QX DNA Size Marker (100 bp–2.5 kb) were included on each run. The repeatability and reproducibility of this method was evaluated by repeated amplification and analysis of four different DNA cheese samples, with both primers for every analysis performed.

### 2.5. Data Analysis and Bioinformatics Processing

Reads were de-multiplexed based on the Illumina indexing system. Following the QIIME pipelines, the USEARCH algorithm (version 8.1.1756, 32-bit) allowed the following steps: chimera filtering; grouping of replicate sequences; sorting sequences per decreasing abundance; and OTU identification, with a species-level taxonomic resolution. When the taxonomy assignment did not reach the species level, the genus or family name were reported. After removing OTUs <5 reads [19], alpha (α) diversity (richness and Shannon indexes), beta (β) diversity (principal component analysis-PCA and principal coordinate analysis-PCoA), and the rarefaction curves were estimated, on the resulting OTU table, by means of R (http://www.r-project.org/index.html; accessed on 7 April 2021), using “vegan” [20] and “agricolae” [21]. Statistical differences (*p* value ≤ 0.05) and evaluation of the influence of the two cheese samplings on the microbial indexes were evaluated by ANOVA followed by the Tukey HSD test. Relative abundance for each OTU across all cheeses was calculated, and “subdominant” and “dominant” OTUs were discriminated according to Zago et al. [18]. Taxonomic analysis was carried out through “reshape2” and “ggplot2” packages [22,23]. RAPD-PCR profiles were imported and analyzed by BioNumerics^TM^ (version 7.6, Applied Maths, Sint-Martens-Latem, Belgium), as previously described [18].

A Neural Network (NN), trained to discriminate GP cheese and HC samples, was implemented with the aim to assess the feasibility of automatic classification for cheese samples and to compare the discriminatory power of metabarcoding and metafingerprinting analyses. The NN was implemented from scratch using Keras python library [24]. The input layer received PCoA data and contained 64 fully connected ReLU units; the second layer had ReLU 32 nodes; the third layer had 16. The last layer had only one sigmoid unit and performed the final binary classification. NN were trained on 80% of the available data, picked as a random stratification (thus maintaining the GP cheese/HC ratio).

Adam optimizer was used to minimize “binary cross-entropy” loss function. The following other performance metrics were measured: Area Under Curve (AUC), true and false positive counts (TP, FP), and true and false negatives count (TN, FN). We also derived Precision, True Positive and True Negative Rates (TPR, TNR), and Accuracy and Binary accuracy. All reported performance metrics were averaged over 10 repetitions of the training process, so as to avoid possible biases due to the random selection of validation set samples. Given the unbalance of tally classes, class weights were computed via the ‘compute_class_weight’ function from the sklearn [25] package and passed to the optimizer.

As a post-hoc investigation, we decided to repeat the NN training tuning class weights so as to explore the limits of the detection power of a hypothetical automated NN-based screening system. Specifically, it measured the possibility to raise True Positive Rate (TPR), i.e., the fraction of HC that were correctly identified, without significantly lowering the True Negative Rate (TNR), i.e., the fraction of GP cheese that were correctly classified. Given that, by definition, there is a tradeoff between TPR and TNR, we fixed the target thresholds to have both metrics higher than 0.9.

## 3. Results

### 3.1. DNA Metabarcoding Analysis

One hundred and nineteen GP cheese samples and 49 samples of generical hard cheeses (i.e., cheeses whose appearance could be confused with Grana Padano PDO cheese, or HC) were analyzed. Overall, 35,842,968 reads were sequenced, with 218,554 reads per sample on average (range 80,636–805,340). A total of 477 OTUs, of which 130 were further split into 48 dominant (≥1% total reads) and 82 subdominant (0.1–1% total reads) taxa, were identified (data not shown).

#### 3.1.1. Species Abundance

**Figure 1:**
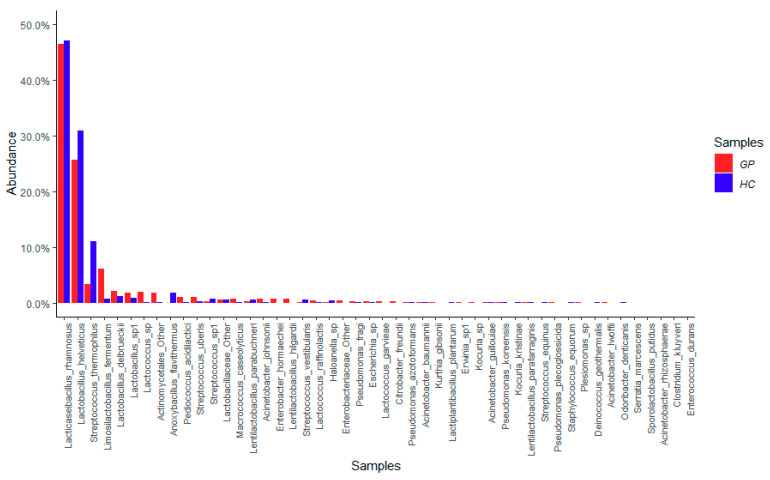
Average values of relative abundance of the 48 dominant taxa retrieved in Grana Padano (GP) and similar hard cheese (HC) samples.

According to the aim of this study, the samples were divided into GP cheese and HC. Considering the relative abundance of the dominant bacterial species (48 taxa; ≥1% total reads) found both in GP and in HC, *Lacticaseibacillus rhamnosus* was the prevalent species, followed by *Lactobacillus helveticus, Streptococcus thermophilus*, *Limosilactobacillus fermentum*, *Lactobacillus delbrueckii, Lactobacillus* spp., and *Lactococcus* spp., with average values between 2% and 46% in GP cheese samples. Notably, the dominant species were less abundant in HC samples compared to GP cheese (Figure 1). In the cheese microbiota, many contaminating bacteria deriving from raw milk or the process environment, such as potentially pathogenic lactic acid bacteria (LAB) (i.e., *Streptococcus uberis* and *Lactococcus garviae*) and other non-LAB taxa (i.e., *Micrococcus*, *Staphylococcus*, *Acinetobacter*, *Pseudomonas,* and many enterobacteria), were also detected (Figure 1).

A different distribution between GP cheese and HC samples of the 82 subdominant species (0.1–1% total reads) was also observed (Figure S1).

#### 3.1.2. Alpha and Beta Diversity

**Figure 2:**
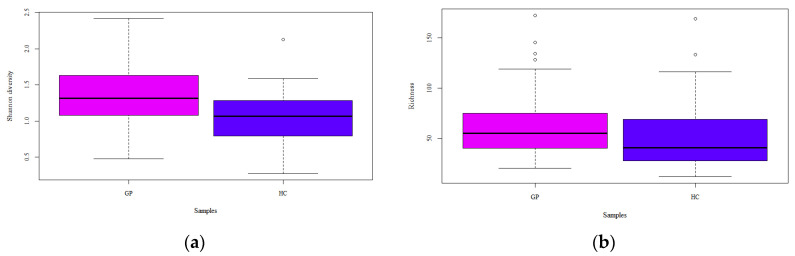
(**a**) Shannon diversity and (**b**) Richness of Grana Padano cheese (GP) and generical hard cheese (HC) samples. Different letters indicate significant (*p* < 0.05) differences between samplings.

**Figure 3:**
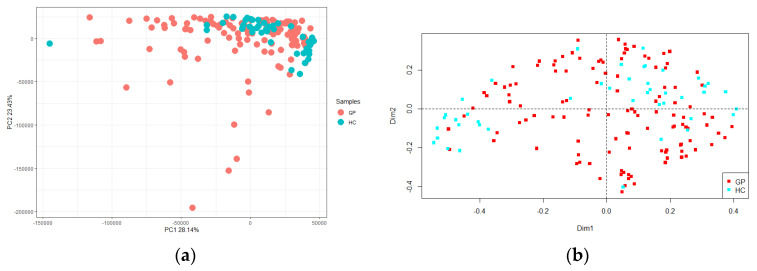
(**a**) Principal component analysis (PCA) and (**b**) Principal coordinate analysis (PCoA) based on operational taxonomic unit (OTU) relative abundance of Grana Padano cheese (GP, red) and generical hard cheeses (HC, green). The first component (horizontal) of PCA accounts for 28.14% of variance, and the second component (vertical) of PCA accounts for 23.43% of variance.

Richness (i.e., the number of different species in each sample) and Shannon indexes are reported (Figure 2). A greater uniformity of species was retrieved in GP cheese, compared to HC, as stated by the statistical significance of the Shannon diversity index (d.f. = 1, F = 16.89, *p* < 0.001), whereas there was not a significant difference in the number of species as indicated by Richness (d.f. = 1, F = 2.02, *p* = 0.157). The PCA showed no differences between GP cheese and HC, which were clustered together (Figure 3a). Conversely, the PCoA reported some differences between GP cheese and HC, as the latter were isolated on both sides of the panel (d.f. = 1, F = 4.70, *p* < 0.001; Figure 3b).

### 3.2. RAPD-PCR Metafingerprinting Analysis

**Figure 4:**
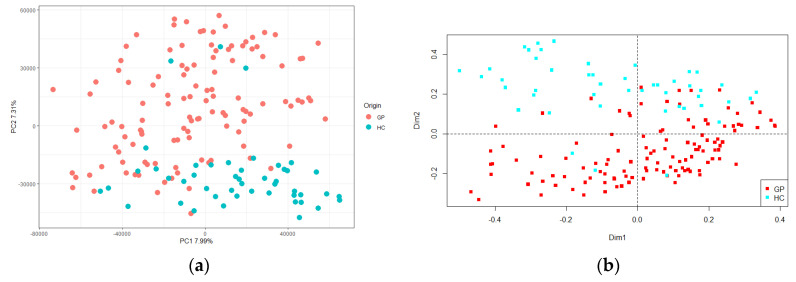
(**a**) Principal component analysis (PCA) and (**b**) Principal coordinate analysis (PCoA) based on band matching and Pearson correlation analysis of the metafingerprinting data of Grana Padano cheese (GP, red) and generical hard cheeses (HC, green). The first component (horizontal) of PCA accounts for 7.99% of variance and the second component (vertical) of PCA accounts for 7.31% of variance.

After RAPD-PCR amplification of the total microbial DNA extracted from all the cheeses, the resulting patterns were analyzed to detect sample-associated profiles and/or specific bands, with a repeatability and reproducibility of 70%. The presence of a panel of shared bands (labelled I in the left side of Figure S2) was highlighted. It included five series of common bands, present in more than 65% of the whole set of samples (GP cheese and HC). Moreover, a panel of unshared bands (or single bands), marked II in the right side of Figure S2, was observed. The matrix obtained from the data was analyzed by PCA, and statistically significant differences were observed between samples (d.f. = 1, F = 3.49, *p* < 0.001), which allowed to clearly separate the GP cheese and HC at the top and bottom of the panel, respectively (Figure 4a). The same data set was subjected to PCoA, and this trend was confirmed (d.f. = 1, F = 18.43, *p* < 0.001; Figure 4b).

### 3.3. Implementation of a Classifier

DNA metabarcoding and RAPD-PCR metafingerprinting PCoA data were used to implement a classifier that could provide a fast, reliable, and automatic categorization of cheese samples into GP cheese or HC. Results for several performance metrics, measured on a randomly picked 20% of the data acting as validation set, are reported in Table 1. The same metrics measured on the training set are reported in Table S1. In general, the metafingerprinting dataset performed better than the metabarcoding dataset, e.g., for Area Under Curve (0.934 vs. 0.657, respectively) and Accuracy (0.928 vs. 0.637, respectively). Metafingerprinting showed a very low average level of False Positives (0.262), indicating that, on average, on about four out of five runs, no False Positives were detected, and in the fifth run, only one GP cheese was erroneously classified as HC.

As a post-hoc analysis, we decided to repeat the NN training on the metafingerprinting PCoA dataset using class weights more skewed toward the detection of HC samples, with the declared target of having both TPR and TNR metrics above the threshold of 0.9. We tested several configurations of weights (data not reported) and found that using an adjustment coefficient equal to 0.25 on the GP cheese class weight, the desired performance was achieved. On the DNA metabarcoding PCoA dataset, no weight adjustment was found to be able to bring both TPR and TNR above the 0.9 target threshold.

## 4. Discussion

Differences in microbial profiling could be useful to establish which microbial components may be responsible for the authentication of PDO cheeses [16,17,26]. On this basis, the microbial diversity of a large sampling that included the entire production area of the GP cheese was investigated and compared with that of 49 generical hard cheeses (HC) taken from retail. Two metataxonomic methods were applied, i.e., 16S rRNA gene sequencing and metagenotyping using RAPD-PCR from total cheese DNA, to identify and implement a robust classifier to differentiate GP cheese from HC. Metataxonomic data revealed a total of about 477 OTUs, including GP cheese and HC, but an overall greater relative abundance in the former ones. This trend occurred among both dominant (among which *Lcb. rhamnosus*, *L. helveticus*, *S. thermophilus*, *Limosilactobacillus fermentum*, *L. delbrueckii, Lactobacillus* spp., and *Lactococcus* spp. prevailed) and subdominant species. While detected LAB taxa within both dominant and subdominant taxa belong to the typical microbiota generally recovered from hard cheeses, the finding of residual psychrotrophic bacteria is not uncommon. Indeed, raw milk for Grana Padano is normally kept under refrigerated conditions before collection and subsequent transport to processing sites [18]. The presence of bacterial DNA of potentially pathogenic LAB and non-LAB taxa is not uncommon, but it has no safety significance, as these bacteria are inactivated by the combined effects of cooking temperature, the curd acidification during the early phases of cheesemaking, and the long ripening times under the harsh conditions (low moisture, low activity water) that characterize the production of hard cheeses [27,28,29]. The Shannon index, which accounts for both microbial richness and evenness, was significantly influenced by the two samplings, highlighting a greater uniformity of species in GP cheese but a similar richness. Differential analysis based on Bray–Curtis dissimilarity between all samples and calculated using species relative abundancies delineated a significant, although incomplete, separation between the two groups.

Cheese production conditions within a PDO area were selected for specific microbial populations. The microbiota of cheeses, especially those (such as Grana Padano) obtained with artisanal processes and from raw milk, plays a fundamental role in defining the qualitative characteristics and safety parameters of the final products. Its composition, structure, and modulation are the result of different selective pressures, e.g., microbiological quality of milk, technology, type of starter used, and processing environment [30]. Therefore, differences in one or more of these factors can be decisive in shaping specific microbiological profiles in cheese. Grana Padano cheese is obtained from raw milk produced in a large production area, which includes most of the provinces of northern Italy included in the Po Valley. Although animal feed is substantially similar, the microbiological composition of milk aimed at GP cheese production can be influenced by management practices at the farm level and by seasonal, climatic, and environmental variations. For example, the presence of some LAB species, such as *Lactiplantibacillus* *plantarum, Lentilactobacillus parabuchneri, Lentilactobacillus parafarraginis, Lentilactobacillus hilgardii,* used also as silage starters, can be related to corn silage fed to cows producing milk for GP [31]. The microbial content of raw milk is subsequently modulated by the selective action of the technology and the practice of using undefined whey starter cultures [32,33]. The greater microbial heterogeneity of raw milk for GP, coming from a large production area, and the ‘balancing’ action exerted by the application of a very similar technology and the addition to raw milk of whey starter cultures, which are usually prepared with comparable methods among the different dairies, are critical to explain the higher species abundance and uniformity observed in GP cheese. On the other hand, HC not being subject to the constraints of a PDO can be produced from milk outside the production area (with its specific microbial content) and with technologies that, although substantially similar, often rely on the use of selected starter cultures and/or the application of thermization, pasteurization, bactofugation, or microfiltration of milk [34]. These selective pressures could be decisive in explaining the lower OTU abundance and species uniformity in HC. The above trends were corroborated, and the separation between the two groups of samples, which was highlighted by the Bray–Curtis dissimilarity test, was noted.

DNA fingerprinting methods, such as RAPD-PCR, are often applied to evaluate the endemicity, or the prevalence, of a given strain. Furthermore, this technique was found useful in discriminating soil microbial communities and estimating their relatedness [35,36]. In a previous study, RAPD-PCR proved useful to highlight a pattern of bands present in all the samples, as well as more specific bands, which aggregated groups of samples, or distinguished single samples, within the microbial community of GP [11]. In the present work, RAPD-PCR was applied to fingerprint the overall bacterial community of GP cheese and HC samples. The obtained metagenotypes were evaluated as possible tools to differentiate the two sampling groups, assuming, unlike metataxonomic analysis, that this technique was able to identify strain- or group-specific differences within complex microbial communities. Data processing of RAPD-PCR profiles using PCA and Bray–Curtis dissimilarity analysis allowed to obtain a clear and statistically very significant separation between GP cheese and HC samples. Metataxonomic and meta-fingerprinting data were then used as inputs to train and validate a two-class (GP cheese vs. HC) classifier based on a neural network as computational model. While metataxonomic data did not allow for reliable classification, the discriminatory power of metafingerprinting enabled to build an extremely robust model (very high binary accuracy). When trained by metagenotyping data, the model correctly classified GP and HC samples. The origin of this differentiation is not currently known, although it is likely that strain-specific peculiarities in the microbial community, determined by previously outlined ecological, geographic, or technological selective pressures, might explain it. The molecular (meta)fingerprint of the entire microbial community could be promising to assist to authenticate GP cheese and to distinguish it from imitation products as part of an attempt to hinder any counterfeits. Further validation will be needed to increase robustness of the classifier and confirm, with unknown samples, its discriminating ability.

## Figures and Tables

**Table 1:** Performance statistics on the validation set for a Neural Network classifier trained on either DNA metabarcoding or RAPD-PCR metafingerprinting PCoA data. The table reports statistics averaged over 10 rounds of training on an 80/20 stratified split of training and validation set. The rightmost column reports metrics when the default class weight for class GP cheese is adjusted by a multiplicative coefficient equal to 0.25, chosen so that both TPR and TNF resulted above a threshold of 0.9.

Statistics	Metabarcoding	Fingerprinting	Fingerprinting Weight Adjusted
FN	3.952	2.188	0.840
FP	8.022	0.262	2.320
TN	15.978	23.738	21.680
TP	5.048	7.812	9.160
AUC	0.657	0.934	0.972
Loss	1.147	0.338	0.312
Precision	0.386	0.968	0.798
TPR	0.561	0.781	0.916
TNR	0.666	0.989	0.903
Accuracy	0.637	0.928	0.907
Balanced Accuracy	0.613	0.885	0.910

FN: number of False Negatives. FP: number of False Positives. TN: True Negatives. TP: True Positives. AUC: Area Under Curve. Loss: final value of loss function, optimized during the training phase. Precision: TP/(TP+FP). TPR, True Positive Rate: TP/(TP+FN). TNR, True Negative Rate: TN/(TN+FP). Accuracy: (TP+TN)/(TP+TN+FP+FN). Balanced Accuracy: (TPR+TNR)/2.

## Supplementary Materials

The following are available online at https://www.mdpi.com/article/10.3390/foods10081826/s1, Figure S1: Average values of relative abundance of the 82 subdominant taxa retrieved in Grana Padano (GP) samples and similar hard cheeses (HC) samples; Figure S2: Band matching cluster analysis showing the relationship between Grana Padano and similar hard cheese samples and M13-/BOXA1R-RAPD-PCR bands; Table S1: Performance statistics on the training set for a Neural Network classifier trained on either metabarcoding or fingerprinting PCoA data.
