# Supplementary material for: Bacterial Community of Grana Padano PDO Cheese and Generical Hard Cheeses: DNA Metabarcoding and DNA Metafingerprinting Analysis to Assess Similarities and Differences
Source: Foods. 2021 Aug 7;10(8):1826. doi: 10.3390/foods10081826 (PMC8392751; doi:10.3390/foods10081826)
Supplement: Supplementary file 1 [file foods-10-01826-s001.zip › Grana_HC, Fig. S1_revised.pdf]

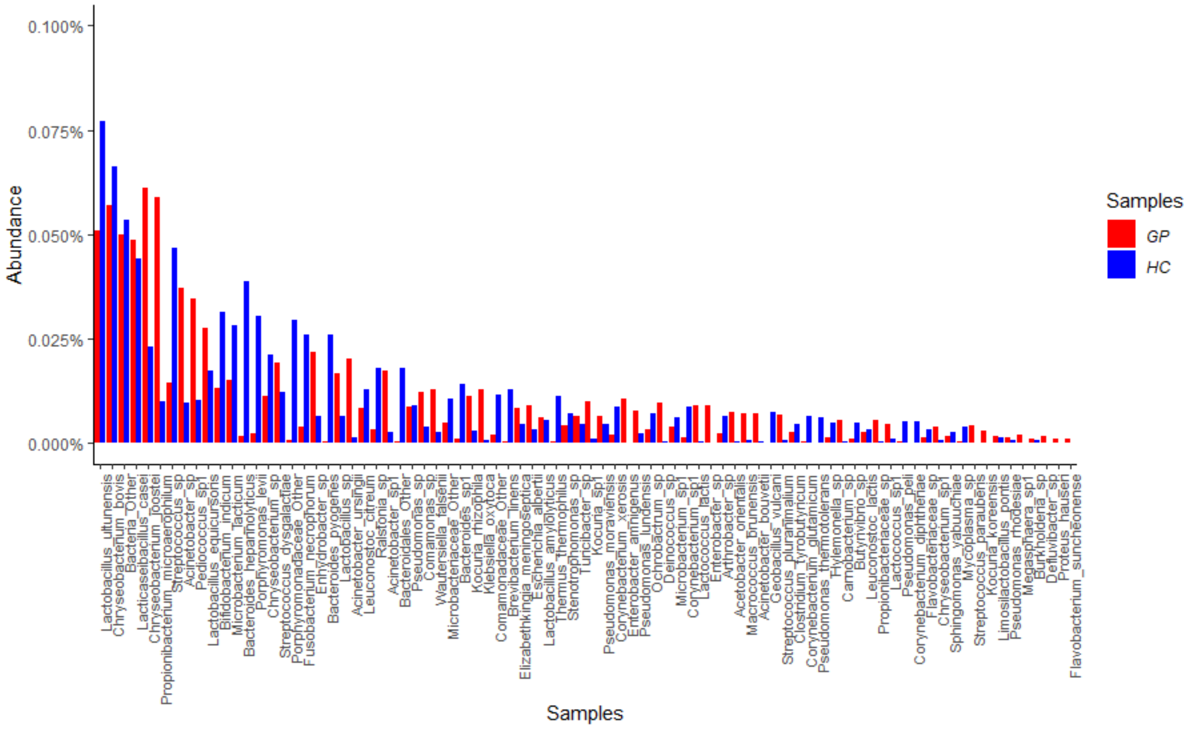

Figure S1. Average values of relative abundance of the 82 subdominant taxa retrieved in Grana Padano (GP) samples and similar hard cheeses (HC) samples.
