# Supplementary material for: Bacterial Community of Grana Padano PDO Cheese and Generical Hard Cheeses: DNA Metabarcoding and DNA Metafingerprinting Analysis to Assess Similarities and Differences
Source: Foods. 2021 Aug 7;10(8):1826. doi: 10.3390/foods10081826 (PMC8392751; doi:10.3390/foods10081826)
Supplement: Supplementary file 1 [file foods-10-01826-s001.zip › Grana_HC, Fig. S2_revised.pdf]

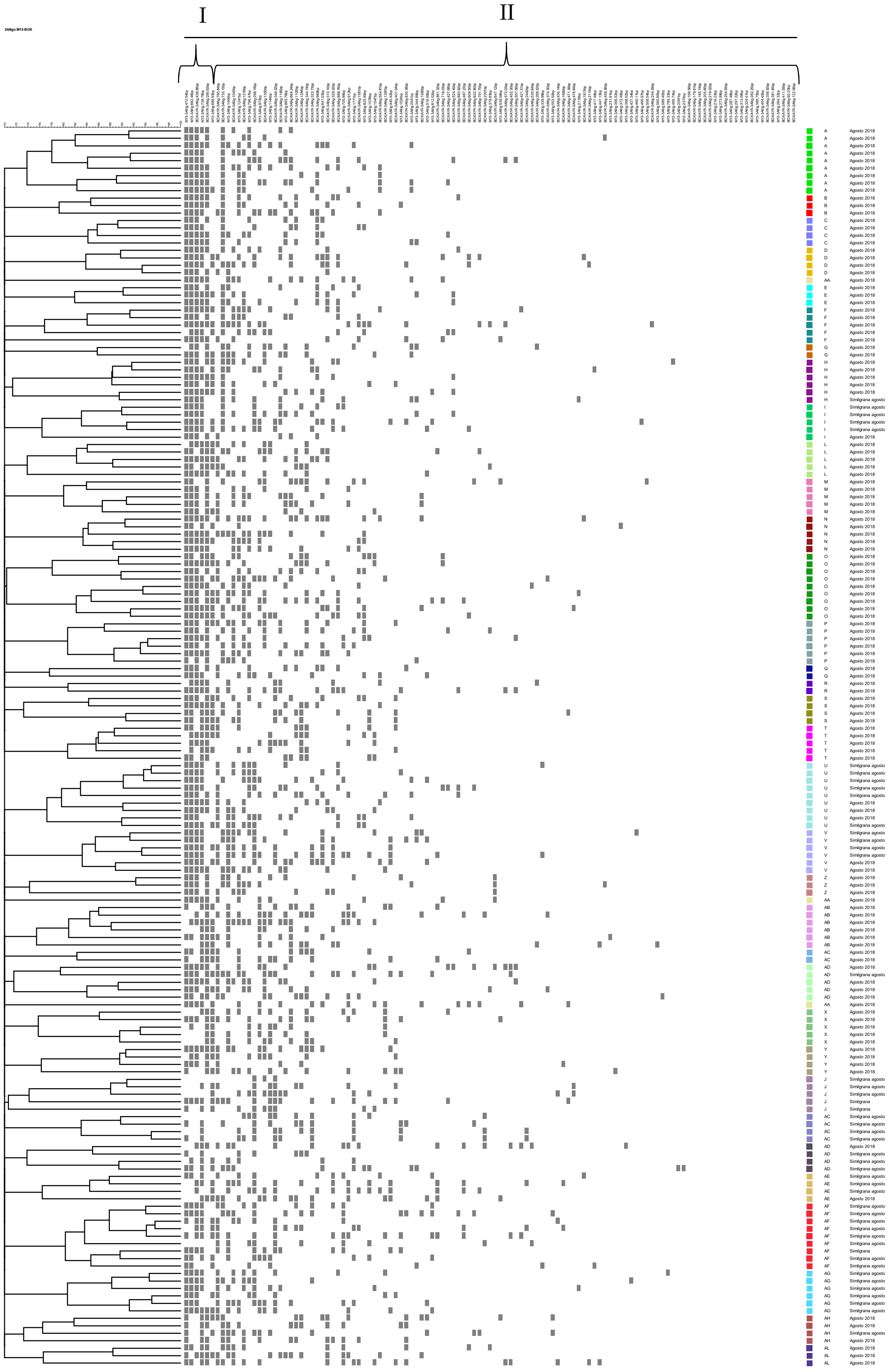

Figure S2. Band matching cluster analysis showing the relationship between Grana Padano and similar hard cheese samples and M13- / BOXA1R-RAPD-PCR bands. Right side: genotype color, genotype name, sample name, region of origin of the sample. Top side: representative bands across the samples. I = core community banding pattern; II = discriminating banding pattern.
